# Is physician implicit bias associated with differences in care by patient race for metastatic cancer-related pain?

**DOI:** 10.1371/journal.pone.0257794

**Published:** 2021-10-27

**Authors:** Kevin Fiscella, Ronald M. Epstein, Jennifer J. Griggs, Mary M. Marshall, Cleveland G. Shields

**Affiliations:** 1 Department of Family Medicine, University of Rochester Medical Center, Rochester, NY, United States of America; 2 Department of Internal Medicine, Institute for Health Policy and Innovation, University of Michigan, Ann Arbor, MI, United States of America; 3 Family and Consumer Sciences, California State University, Long Beach, CA, United States of America; 4 Department of Human Development & Family Studies, College of Health and Human Sciences, Purdue University, West Lafayette, IN, United States of America; Osakidetza Basque Health Service, SPAIN

## Abstract

**Rationale:**

Implicit racial bias affects many human interactions including patient-physician encounters. Its impact, however, varies between studies. We assessed the effects of physician implicit, racial bias on their management of cancer-related pain using a randomized field experiment.

**Methods:**

We conducted an analysis of a randomized field experiment between 2012 and 2016 with 96 primary care physicians and oncologists using unannounced, Black and White standardized patients (SPs)who reported uncontrolled bone pain from metastatic lung cancer. We assessed implicit bias using a pain-adaptation of the race Implicit Association Test. We assessed clinical care by reviewing medical records and prescriptions, and we assessed communication from coded transcripts and covert audiotapes of the unannounced standardized patient office visits. We assessed effects of interactions of physicians’ implicit bias and SP race with clinical care and communication outcomes. We conducted a slopes analysis to examine the nature of significant interactions.

**Results:**

As hypothesized, physicians with greater implicit bias provided lower quality care to Black SPs, including fewer renewals for an indicated opioid prescription and less patient-centered pain communication, but similar routine pain assessment. In contrast to our other hypotheses, physician implicit bias did not interact with SP race for prognostic communication or verbal dominance. Analysis of the slopes for the cross-over interactions showed that greater physician bias was manifested by more frequent opioid prescribing and greater discussion of pain for White SPs and slightly less frequent prescribing and pain talk for Black SPs with the opposite effect among physicians with lower implicit bias. Findings are limited by use of an unvalidated, pain-adapted IAT.

**Conclusion:**

Using SP methodology, physicians’ implicit bias was associated with clinically meaningful, racial differences in management of uncontrolled pain related to metastatic lung cancer. There is favorable treatment of White or Black SPs, depending on the level of implicit bias.

## Introduction

There is evidence that implicit racial bias affects many human interactions including patient-physician encounters [1]. Implicit bias may undermine health care provided to minority patients [2,3]. These effects may be mediated through verbal and non-verbal communication and clinical decision-making [3]. For example, there is robust evidence that Black patients are less likely to receive opioid prescriptions for pain compared with White patients [4,5]. Implicit racial bias reduces empathetic related brain activity towards the apparent pain experienced by an individual of a different race [6–8] suggesting a potential mechanism through which implicit bias might reduce prescribing opioids for pain for Black patients.

Real world observational studies generally report that physician implicit bias adversely impacts health care for Black patients [3]. In contrast, studies of physician implicit bias involving experimental vignette studies have often shown no effect on clinical decisions [9]. Standardized patients (SPs), i.e. actors trained to portray patients (sometimes referred to as “secret shoppers”), offer a unique window into clinical interactions in that patient presentations can be standardized, demographics and patient presentations can be manipulated, and SPs can be deployed with high degrees of fidelity so that they escape detection [10]. With the exception of audit studies for new patient appointments [11], we are not aware of real world experimental studies involving Black and White SPs; although such studies have been used to assess racial bias outside of health care, for example, in hiring decisions [12,13].

We designed the current study to assess whether the observational findings from real world studies would extend to a randomized field experiment. Specifically, we aimed to examine the role of physician implicit bias in actual practice through the use of a randomized field experiment in which unannounced SPs visited consenting physicians in their offices. The SPs portrayed patients with advanced cancer; the four SP scenarios differed only by race (Black or White) and by role (typical or activated) but otherwise had identical scripts and achieved high fidelity. We previously examined typical versus activated roles because there is evidence that when patients are activated implicit biases may be attenuated [14,15]. Activated patients are more likely to make their desires known through a more assertive explanation of their problems and by asking their physicians questions. However, as previously reported, this hypothesis was not confirmed, i.e. activation did not attenuate SP race effects on opioid prescribing [16].

In this study, we examined the interaction between physicians’ implicit bias and patients’ race on the physician management of a new patient who presents with previously-diagnosed metastatic lung cancer and bone pain uncontrolled by current doses of opioid medications. Based on real world observational studies of the effects of physician implicit bias on care [3] and the presumed adverse impact of implicit bias on cancer care [17], we hypothesized that interactions between physician implicit bias and patient race, i.e. physicians with higher implicit bias seeing Black SPs would tend to provide less optimal care [18]. Specifically, we hypothesized that greater physician bias would be associated with lower likelihood of renewing opioid prescriptions when the SP was Black. We further hypothesized that physicians with greater implicit bias would use less patient-centered pain communication (frequency and depth of physician responses to and exploration of patients’ expressions of concerns about pain) [19–21] and less prognosis communication among Black SPs [22,23]. Based on prior observations [21,24–26], we hypothesized that physicians with greater implicit bias would display greater verbal dominance with Black SPs. Based on prior reports that disparities in routine communication generally are absent or small [27–29], we anticipated that implicit bias would *not* be associated with differences in routine pain assessment (e.g. questions about onset, character, location, radiation, etc.) by SP race.

## Materials and methods

### Overview

**Fig 1:**
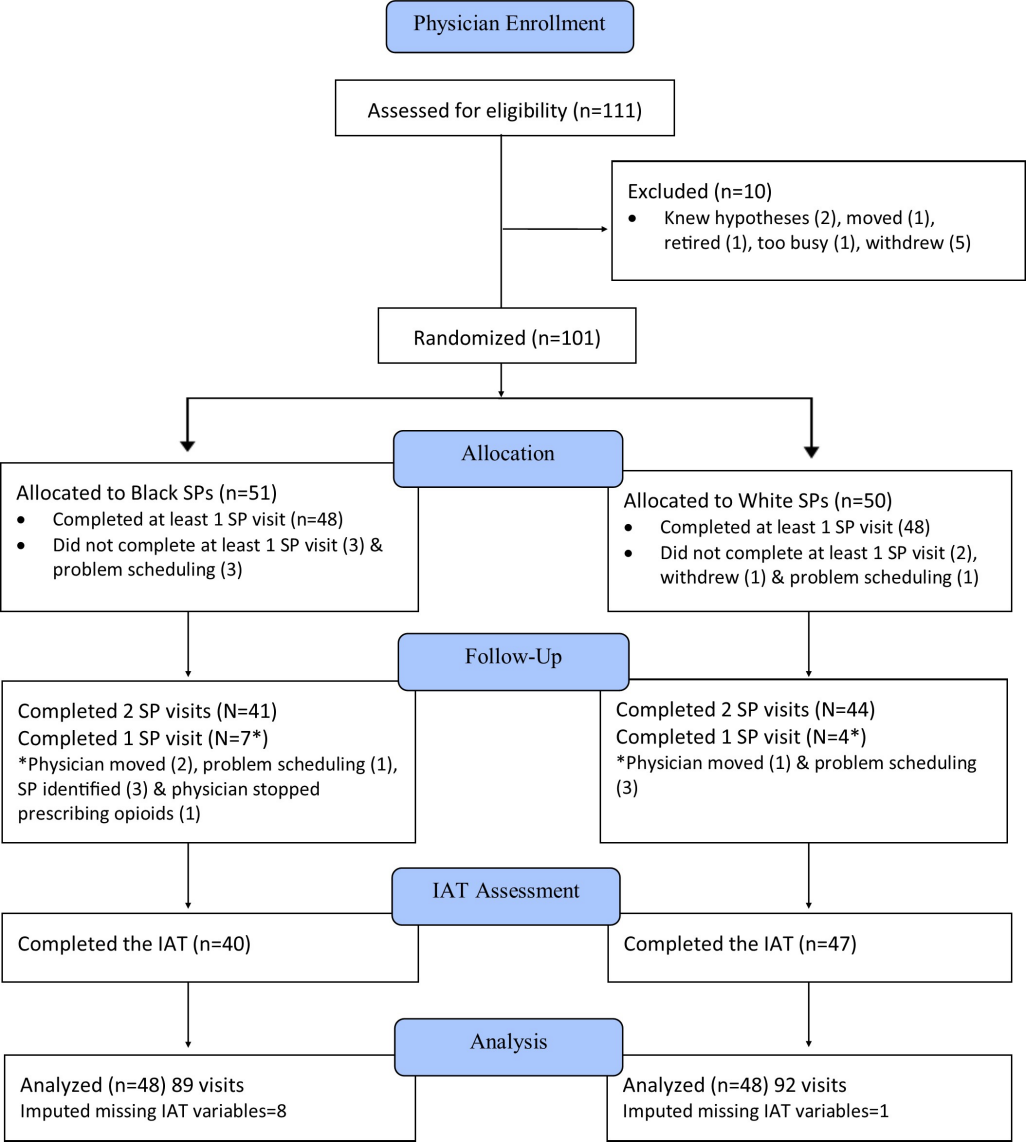
Participant flow diagram.

We conducted an analysis of a randomized field experiment in 2012 through 2016 on the association of physician implicit attitudes on care provided to Black and White patients. The study team trained Black and White SPs for office visits at primary care and oncology practices in New York, Indiana and Michigan. Each study site received approval in conformance to the Declaration of Helsinki guidelines, i.e. Purdue University Institutional Review Board (1009009643), the University of Rochester Research Subjects Review Board (RSRB00033086), the University of Michigan Human Research Protection Program (HUM00067842) and the McLaren Health Care Corporation Human Research Protections Program (2014–00098). Details of the study have been published [18]. Fig 1 shows participant flow through the study.

### Physician participants

Study investigators approached oncologists and primary care physicians individually to identify those willing to participate. In the US, primary care clinicians often provide symptomatic treatments for patients with cancer and refer patients with cancer to oncologists for anti-cancer treatments. Physicians agreed to participate in two, unannounced SP visits, a hidden audio recording of the SP visits and to respond to surveys, including a computer administered Implicit Associations Test (IAT). Physician demographics, including age, sex, specialty and race/ethnicity, were based on physician self-report. All study participants provided written, informed consent.

### Standardized patients

The research team hired and trained SPs at each site to assess physician prescribing and communication. As described above, the four SP roles created were identical except for self-identified (and apparent) race and role: 1) a Black activated SP, 2) a Black typical SP, 3) a White activated SP, and 4) a White typical SP. The investigators trained SPs to describe a history of advanced lung cancer treated with radiation and to say they recently moved from another state to be closer to an adult child. Detailed medical records were created to provide imaging, pathology, and treatment reports as well as physician notes. These were sent to physician offices prior to SPs office visits.

The SP role included spontaneous reports of uncontrolled pain despite exceeding the prescribed doses of pain medication. The research team trained some of the SPs to assume an activated patient role [21,30]. This SP role included asking more direct questions about their diagnosis and treatment including pain management, asking for clarifications and redirecting the discussion when their concerns were not addressed. SPs were blinded to the study hypotheses about race, activation and implicit bias. SP visits were audio-recorded and transcribed to assess SP-physician communication. Seventeen (17) different individuals over the three sites were trained as SPs and underwent non-invasive, office care, e.g. they were trained to defer rectal examinations, blood tests and imaging.

### Randomization and blinding

The statistician for the primary study randomly assigned each physician stratified by specialty to visits by two Black or two White SPs, one activated and one not. The sequence for these office visits (activated SP followed a few months later by a typical SP or vice versa) was also randomly assigned within each physician. Coders were blinded to SP race, role and study hypotheses. Physicians were blinded to study aims including the fact that SPs would be of different races.

### Implicit bias measure

After completion of all SP visits, we used a pain adaptation of the race IAT to assess physicians’ implicit racial bias once, using the IAT, which is a computer-supported measure of implicit associations, often based on images of people of different races and words denoting good or bad attributes. The premise behind the IAT is that people more quickly pair concepts they associate with each other than concepts with weaker associations. The IAT has been used extensively to assess implicit racial associations [31–33]. The IAT predicts intergroup behavior independently of measures of overt racial attitudes in both laboratory and field settings [34]. Participants matched words and Black and White facial images, both pained and non-pained expression as quickly as possible using two keys (“i” or “e”). More detailed descriptions of the IAT are available [35]. Using the algorithm of Nosek et al [35], we calculated the standardized differences between incompatible versus compatible word and picture pairings for Black and White facial images, whether pained or not. IAT was calculated as the mean of the standardized differences with a higher score indicating greater implicit bias for the participant. We used a SAS macro to assess the scoring algorithm endorsed by the Greenwald et al [36]. The overall IAT showed good internal reliability (alpha_full_ = 0.74, alpha_nopain_ = 0.72, alpha_pain_ = 0.72). See supporting information file S1 Table. Our pain adapted version of the IAT is publically available (https://osf.io/2bpe4/).

### Physician opioid pain prescribing measures

We obtained copies of study physicians’ medical records and prescriptions, determined whether an opioid was prescribed and created a dichotomous variable to indicate whether or not an opioid was prescribed.

### Physician office visit communication measures

Physicians consented to covert audio recordings of SP visits but were not given any information about the demographics of the SPs, nature of the role, or the timing of the visit. Audio-recordings were professionally transcribed, stripped of any racial identifiers and coded by the research team. We used the intraclass correlation coefficient (ICC) to calculate interrater reliability of the coded communication variables using a SAS macro [37].

#### Patient-centered pain assessment

Research assistants coded the transcripts using the Measure of Physician Pain Assessment (MPPA), which captures frequency and depth of physician responses to and exploration of patients’ expressions of concerns about their pain (e.g. “I understand your medication is not controlling your pain”). This measure was previously developed, piloted and validated for use in outpatient cancer consultations [38].

#### Routine pain assessment

Using the same methods, we also coded for routine pain assessment (e.g. standard questions regarding pain location, intensity, character, etc.). Coders noted the presence of pain assessment and patient centered pain discussion items that we then summed for a total prognosis score. Scores on each item were truncated at four so that no one item dominated the total prognosis score.

#### Prognosis-related discussion

We assessed prognosis and treatment choice discussions using the Prognostic Treatment Choices Scale (PTCC), which was developed in a pilot study [39] and recently used in a large randomized intervention trial to improve communication in patients with advanced cancer [40]. These items assess physicians’ communication of diagnostic and prognostic information and treatment options with patients with metastatic disease. Sample items are, “Physician asks if patient wants to know more about the diagnosis” and “Assessing if patients understand their diagnosis.” Coders noted the presence of prognosis items, which were then summed for a total prognosis score. Scores of each item were capped at four so that no one item dominated the prognosis total score.

#### Physician verbal dominance

We examined physician verbal dominance during the visit in three ways: physician use of “I” statements, conversational dominance and physician conversational cutoffs. We assessed physician word choice using the 2015 version of the Linguistic Inquiry and Word Count (LIWC) software [41]. LIWC categorizes words in transcripts by types of speech, nouns, verbs, pronouns and other categories. We used the software to calculate the use of first person singular pronouns by the physician and to calculate total physician word count. We assessed higher frequency of physician “I” statements, which is the percentage of total words spoken by physicians [42]. We also used physician proportion of total words exchanged during the visit as a second measure of conversational dominance [43]. Last, we used rater coding of physician topic cutoffs, i.e. abrupt change of topic from what the patient was discussing [44,45].

### Statistical analysis

Survey and other data collected on paper were double entered into MS Excel or Qualtrix^TM^ files, which we imported into SAS 9.4 (Cary, NC) for data processing. Data management and variable creation were done in SAS 9.4 (Cary, NC). Data analysis was conducted in STATA 15 (College Station, Texas).

We used mixed-effects linear regression and mixed-effects logistic regression to estimate the effects of race and the role of SPs and physician implicit bias on the clinical or communicational outcome. We included a random intercept in these models to account for the nesting of multiple visits within physicians [46]. We operationalized IAT as a continuous variable and included interaction terms with SP race and SP role. We examined the statistical significance of slopes and cross over interactions for Black and White SPs related to physician IAT scores. We included controls within each model for type of physician (oncologist or PCP) and research site (site = 1 if last site) and SP role. We used recommended strategies for managing missing data by using STATA missing data protocols for regression and logistic regression analyses [47]. Missingness was not associated with any physician demographic variables. Physicians who did not complete the second SP visit had missing values on IAT, but that was the only relationship. Reasons for failure to complete the second SP visit included physician relocation and declines when the office was too busy to schedule another visit. Missing data were treated as missing at random. We report estimates (β/OR and confidence intervals), ICC, log ratio test and deviance for modeling goodness of fit.

We employed multiple imputations with STATA mixed and meqrlogit procedures using the multiple imputation command [48] to address missing data and compared results with a complete case analysis. We conducted several additional sensitivity analyses. We added physician race as a covariate in the models. We substituted use of “We” for “I” statements [49]. Excluding visits in which SPs were detected did not alter our findings nor did controlling for detected visits as covariates change our findings. Finally, we calculated the IAT for pain faces and non-pain faces separately and examined the interaction of IAT with SP race.

## Results

SPs completed 183 visits with 90 physicians. After prompting, physicians were able to identify SPs in 15% of office visits. Descriptive data appear in Table 1. Approximately 40% of the physician sample were female, 64% were White, and the mean age was 52 years (range 29–81). Eighty-three physicians (86%) completed the IAT, giving us 159/183 (87%) SP visits with raw IAT scores (Table 1). We addressed missing values using an imputed data set. Among physicians completing the IAT, 47% percent were oncologists, 53% were primary care physicians, roughly 40% were female, 58% were White, 15% Black, 7% Hispanic and 22% South Asian with an average age of 51 years (range 29–76). The mean length of visits was 36 minutes. Visit duration did not differ by physician IAT score, SP race, or physician specialty. The intraclass correlations (ICCs) for patient-centered pain assessment, prognosis related discussion and routine pain assessment in this sample were 0.73, 0.75, and 0.85 respectively.

**Table 1:** Physician characteristics by the race of the SP.

Variable		SP Race	
	SP Visits (n = 181)	White (n = 92)	Black (n = 89)
***Physicians* (n = 90)**			
Mean Age years (SD)	51.9	50.3 (1.42)	53.3 (1.32)
% Female	39%	37%	41%
% White	67%	71%	62%
% PCPs	55%	54%	56%
% Black	19%	18%	19%
% Hispanic	8%	4%	11%
% South Asian	22%	23%	18%
% Other	12%	7%	16%
	*Percentages do not add to 100% because 20 physicians identified with more than one race*		
Mean non-imputed IAT Score* (SD) n = 159	0.89 (0.53)	0.79 (.53)	1.02 (.53)
***Opioid Prescribing***			
% No Opioid Prescription Given	26%	22%	30%
***Physician Verbal Dominance***			
% Physician Mean I-Words** (SD)	3% (0.94)	3% (0.95)	3% (0.94)
Mean Physician Cut Off*** (SD)	54% (0.82)	47% (0.76)	62% (0.89)
% Physician Total Words**** (SD)	68% (0.12)	69% (0.10)	67% (0.13)

SP = standardized patient, SD = standard deviation, PCP = Primary Care Physician, IAT = Implicit Association Test.

Percent (%) of primary care physicians relative to all physicians, i.e. primary care + oncology physicians.

*The IAT Score is the mean difference in latency between compatible versus incompatible matching of words and faces to Black vs White actors. Higher scores indicate greater response latency differences and greater implicit bias. Range in IAT Scores was -0.797 to 1.801.

** % I-Words = number of I-words (I, me and mine) uttered by the physician, divided by the total number of words uttered by the physician.

*** Cut Off = abrupt change of topic from what the patient was discussing. Score is the percentage of visits containing at least one Cut Off.

****Percent Physician Total Words = the number of total words uttered by the physician, divided by the number of words uttered by all parties in the visit.

Our hypotheses focused on interactions between physician IAT score and SP race. However, we first present the statistically significant, non-interaction effects shown in Table 2A and 2B.

**Table 2a:** Patient care outcomes: SP race, IAT, and interaction of race and IAT^1^.

Outcome	Routine Pain Assessment		Use of I-Statements		Physician Cut Off		Physician % Words	
	β	CI	β	CI	OR	CI	β	CI
Black SP (vs. White SP)	1.341	-2.137, 4.821	.186	-.301, .804	.566	.107, 2.990	0.038	*-*.043, .119
IAT	**.195**	-2.312, 2.702*	**.498**	.027, .969*	1.302	.345, 4.780	0.045	*-*.015, .106
Race X IAT	-1.450	-4.638, 1.720	-.156	-.757, .445	2.131	.435, 10.446	-0.073	-.150, .004
Activated SP (vs non-activated SP)	-1.580	-2.026, 2.177	**.938**	.502, 1.373*	1.989	.478, 8.271	-0.028	-.084, .027
Activated X IAT	-.098	-2.067, 1.870	**-.493**	-.909, -.076*	.561	.434, 2.187	-0.02	-.074, .032
ICC	.474		.327		.208		.*362*	
Log ratio	10.370		6.106*				*9*.*630*	
Deviance for goodness of fit	58.204		39.495		202.616		27.481	

^1^ All analyses included covariates for research site, physician age, and physician specialty (PCP or oncologist). S2 Table (supporting information) shows the full models with the key outcomes.

**β/OR** = Regression Coefficient/Odds Ratio, SP = standardized patient, etc. Bolded results fall outside of the 95% confidence intervals.

**Table 2b:** Patient care outcomes: SP race, IAT, and interaction of race and IAT^1^.

Outcome	Prescription of an Opioid		Patient-Centered Pain Talk		Patient-Centered Prognosis	
	OR	CI	β	CI	β	CI
Black SP (vs. White SP)	1.903	.356, 10.163	2.668	-1.472, 6.807	0.609	-.391, 1.610
IAT	4.252	.788, 22.920	2.989	-.174, 6.52	0.307	-.433, 1.047
Black Race X IAT	**.113**	.*064*, .783*	**-4.163**	-8.113, -.211*	-.727	-1.681, .228
Active SP (vs. Typical SP)	1.301	.270, 6.267	**6.144**	3.043, 9.244*	.189	-.472, .851
Active X IAT	2.514	.507, 12.456	-2.320	-4.164, .648	-.252	-.886, .381
ICC	.*274*		.*245*		.*372*	
Log ratio			*3*.*96*		*10*.*37*	
Deviance for model goodness of fit	127.612		60.995		45.122	

^1^ All analyses included covariates for research site, physician age, and physician specialty (PCP or oncologist). S2 Table (supporting information) shows the full models with the key outcomes.

**β/OR** = Regression Coefficient/Odds Ratio, SP = standardized patient, etc. Bolded results fall outside of the 95% confidence intervals.

Physicians provided more patient-centered talk to active SPs than typical SPs; physicians with high IAT scores made fewer I-statements with active SPs than typical SPs. Physicians with higher IAT scores used more “I” statements in general and provided more routine pain assessment (Table 2A). Activated SPs received more patient-centered pain talk and physician “I” statements, presumably in response to more questions (Table 2A).

**Fig 2:**
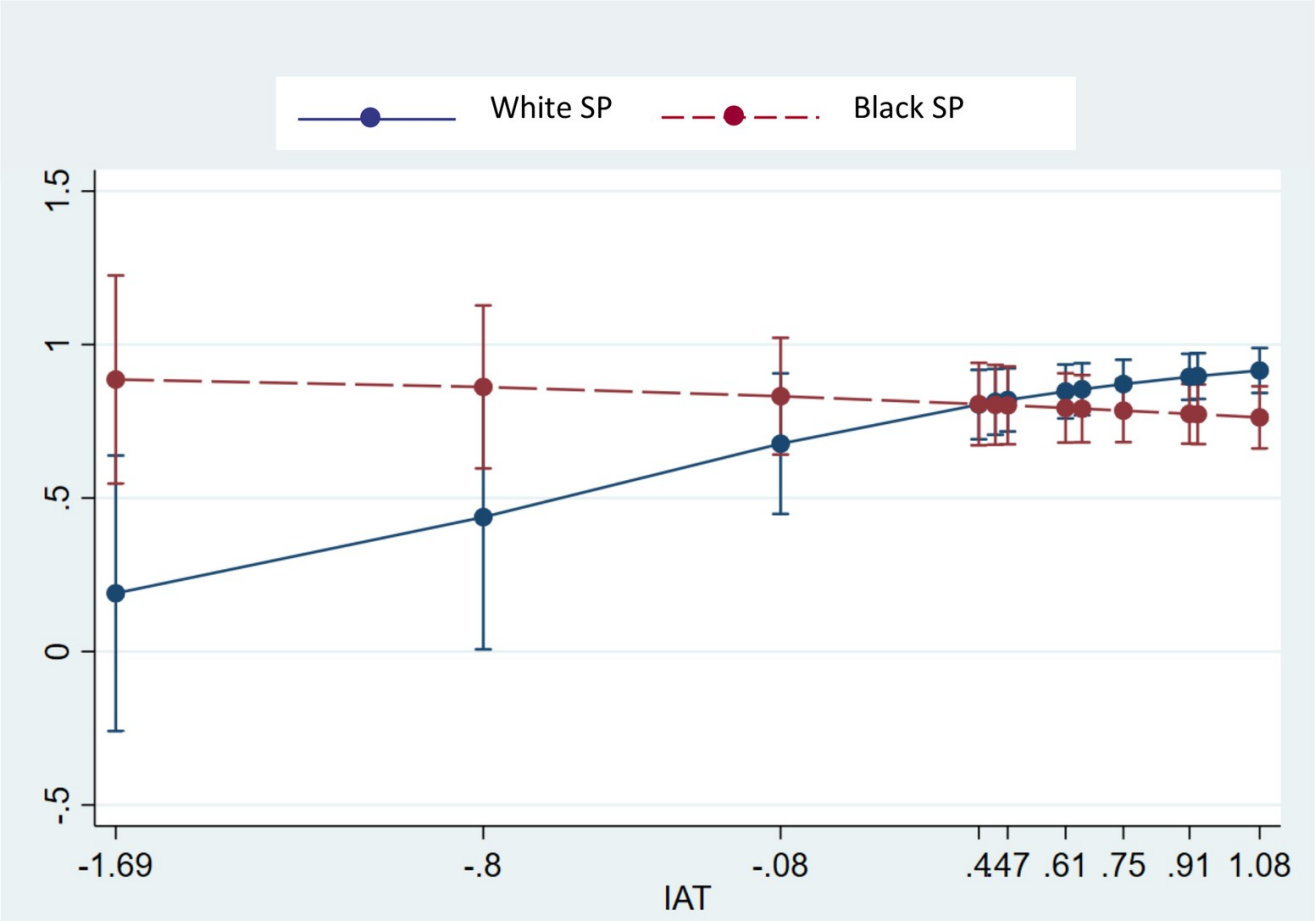
Opioid Rx given by race & IAT.

We hypothesized that greater physician bias would be associated with lower likelihood of renewing opioid prescriptions. This is supported by the results (Table 2B). The interaction of race and IAT was associated with lower odds (odds ratio) of renewing opioid prescriptions. We explored the slopes of this cross-over interaction (Fig 2). For Black SPs, a one-unit increase in IAT was associated with a 47% non-statistically significant decrease in the odds of receiving an opioid prescription. Notably, for White SPs, a one-unit increase in IAT was associated with a *376% increase in the odds of opioid prescription*, but this did not attain statistical significance (p = .08). Among Black and White SPs seen by physicians with lower IAT scores, we observed the opposite relationship with Black SPs receiving more opioid prescriptions than Whites. Notably, the slope for IAT and opioid prescription is significantly different between White SPs and Black SPs, OR = .11 or OR = 8.90 depending on the reference group (p = .029).

We hypothesized that patient-centered communication about pain and prognosis would be lower for Black SPs when physicians had higher IAT scores. This hypothesis was confirmed for patient centered pain communication. We examined the slopes for this cross-over interaction. We found that for Black SPs, a one-unit increase in IAT is associated with a non-statistically significant decrease in the slope pain talk. For White SPs, a one-unit increase in IAT is also non-statistically significant but trending towards improved communication for White SPs. Similar to the findings for opioid prescribing, opposite relationships were observed among physicians with lower IAT scores with these physicians providing greater patient-centered pain talk to Black than White SPs. Notably, the slope for IAT and opioid prescription is statistically significantly different between White SPs and Black SPs, β = 4.09 or β = -4.09 (p = .029) depending on the reference group.

Our hypothesis that prognosis communication would interact with physician bias was not confirmed. Our hypothesis that routine pain assessment would not be associated with race and IAT was supported by our findings. Our hypothesis that physicians with higher IAT scores seeing Black SPs would be more verbally dominant was not confirmed. We did not hypothesize any interactions for activated SPs.

**Fig 3:**
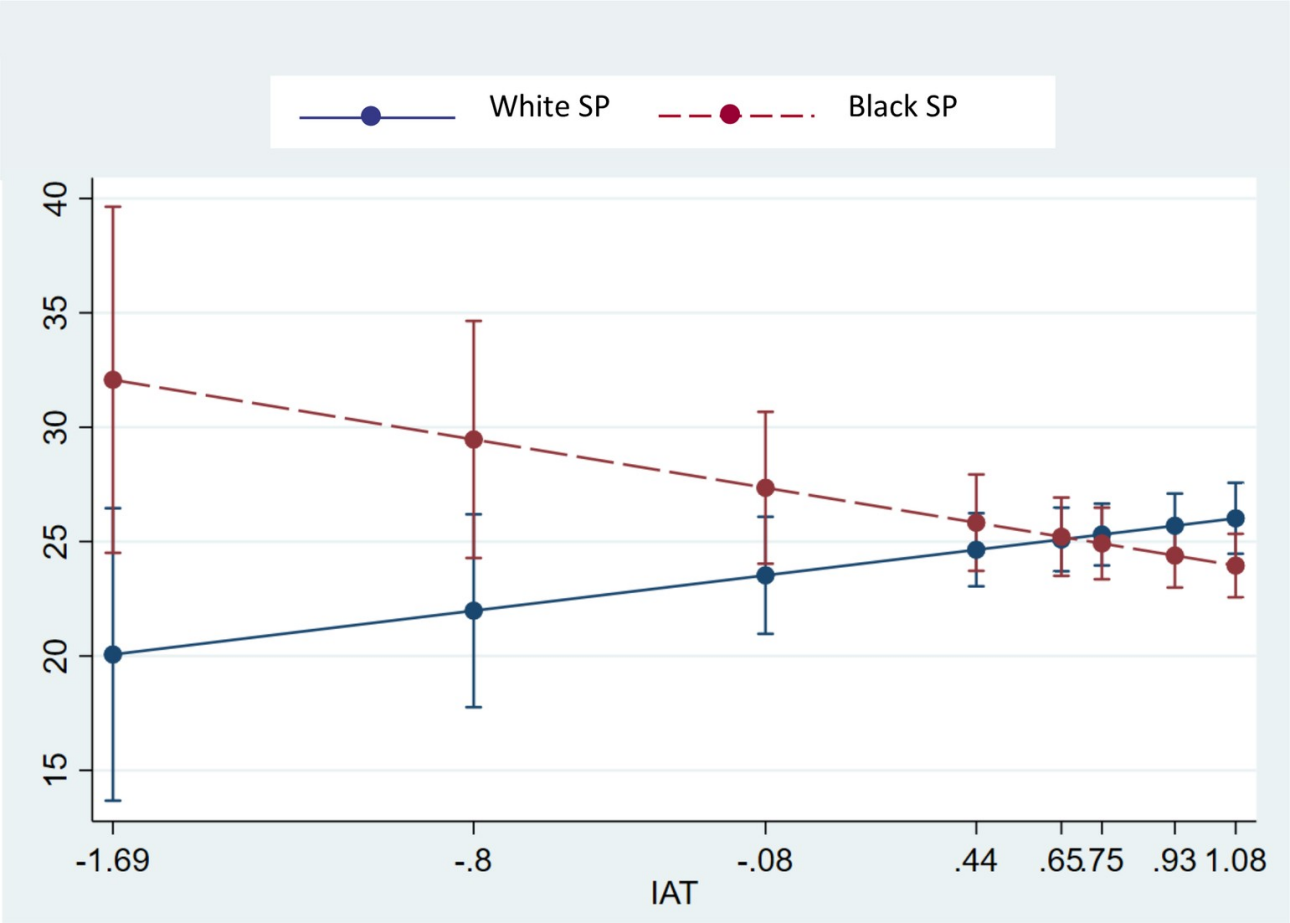
Interaction of IAT & race on PCC pain talk.

We conducted several sensitivity analyses. A complete case analysis yielded similar results. When we added physician race as a covariate in the models, the results were unchanged. Substitution of the use of “We” for “I” statements showed no interaction with IAT and SP race. Excluding visits in which SPs were detected did not alter our findings nor did controlling for detected visits as covariates change our findings. We assessed the IAT scores for pain faces and non-pain faces separately and examined the interaction of IAT with SP race. S1 Table in the supporting information files shows the comparison of the coefficients and 95% CI’s of these interactions. None of the pain or non-pain coefficients were significant while the interaction of IAT and SP race were significant for opioid prescribing and patient centered pain talk (Fig 3).

## Discussion

Findings from this field study involving randomized assignment of Black and White SPs to primary care and physicians and oncologists, are generally consistent with those from observational studies of practicing physicians. We confirmed hypotheses related to interactions between physicians’ implicit bias and SP race for opioid prescribing and patient-centered communication about pain. Both of these interactions were driven by physicians with higher bias favoring White SPs and disfavoring Black SPs. The opposite relationships were observed among Black and White SPs who were seen by physicians who showed lower implicit bias. In these instances, Black SP were more likely to receive opioid prescriptions and receive more patient-centered pain communication than White SPs. As predicted, we did not observe such interactions for routine pain assessment.

In previous observational studies, higher implicit bias was often associated with lower quality of care for minority patients [3], including patient ratings of patient centered care [17,50], shorter visits [17] and physician verbal dominance [26,49,51]. These types of observational studies are limited by unmeasured confounding and nuances in clinical context. In contrast, previous experimental studies of implicit bias, which involved patient vignettes, have generally yielded null findings with only two of nine studies reporting statistically significant relationships between implicit bias and clinical decision-making [9]. The reasons for these conflicting findings with real world observational data are not clear but could plausibly involve differences in activation of physician rational-cognitive process in vignettes compared with reliance on more implicit processes in the real world [52]. Moreover, physicians’ racial stereotypes regarding Black patients having higher pain tolerance might also contribute to under prescribing for pain in real world settings [53,54]. Importantly, our findings suggest that greater physician bias is manifested by not just less favorable treatment of Black SPs, but that higher implicit bias is associated with more favorable treatment of White SPs. This finding is consistent with studies documenting the effects of in-group favoritism [55] and highlights the role of implicit bias on both favoring White patients and disfavoring Black patients.

We did not hypothesize higher prescribing of opioids and more patient-centered care for pain communication for Black SPs among physicians with less implicit bias. Previous studies suggest that some Whites cope with anxiety related to inter-racial interactions by over-compensating with Blacks in an effort to show they are not biased [56–58]. Possibly, this over-compensation accounts for this unexpected finding. However, we are not aware of empirical data suggesting this occurs more often among those who score lower on the IAT. This unexpected finding requires replication.

Several hypotheses were not confirmed. Physician implicit bias did not interact with physician “I” statements, prognosis talk, physician word count, as a percentage of total word count during the visit, or quick changes in topic (cut-offs). We cannot be sure if these findings reflect differences in communication patterns between real and SPs and their physicians or whether previous observational findings reflect unmeasured confounding [26,49]. While our standardization of patient behavior allowed us to isolate the effect of patient race, this decontextualizing of race likely masked the real world complexity of interactions between physicians and actual patients of different races. Further study is necessary to examine this phenomenon with real patients.

The strengths of our study include the use of an experimental design involving unannounced, White and Black SPs making office visits to primary care physicians and oncologists. Rates of physician detection of these SPs were low, suggesting that physicians treated the SPs similarly to actual patients. To our knowledge, this is the first study to use a randomized field experiment to assess the effect of physician bias on care provided to Black and White patients in any clinical context.

There are several important limitations. We trained SPs for one specific context—a new, male patient with metastatic lung cancer and pain inadequately controlled by opioid analgesics. We randomized physicians to either two Black or two White SPs thus precluding assessing of within-physician effects. We included both primary care physicians and oncologists, which, while increasing generalizability, may have masked specialty differences that our study was underpowered to detect. We used a pain adaptation of the IAT that has not been validated in published studies. It is possible that White physicians are less familiar with Black faces expressing pain compared with White faces expressing pain. However, analyses that excluded use of the pained Black and White expression showed similar results. We cannot exclude the possibility that IAT is a marker for other unmeasured physician characteristics. Further, we did not control for multiple comparisons in order to avoid false negative findings resulting from our modest sample. Last, a meta-analysis showed that IAT effect sizes were smaller when variants of the IAT and when the outcome involved resource allocation [34]. These limitations underscore the need for replication of these findings using SP methodology.

## Conclusions

We observed that physician implicit bias is associated with selected, clinically meaningful, racial differences in care among new patients with uncontrolled pain related to metastatic lung cancer. Physicians’ favorable treatment to White or Black SPs depended on the level of implicit bias. Our findings have implications for clinical care and for monitoring of racial disparities in care. Our findings provide further evidence that physician implicit bias can affect clinical care provided to Black and White patients while underscoring the need for research on mitigation strategies [59] and further exploration of the role of low implicit bias in favoring Black patients. These findings also provide further justification for systems for monitoring racial disparities in health care and for deploying best practices for mitigating them [60–64].

## Supporting information

S1 Table Difference in prescribing between black and white standardized patients at different levels of IAT. (DOCX)

S2 Table Results based on full IAT, non-pain IAT and pain IAT. (DOCX)
