# Supplementary material for: Is physician implicit bias associated with differences in care by patient race for metastatic cancer-related pain?
Source: PLoS One. 2021 Oct 27;16(10):e0257794. doi: 10.1371/journal.pone.0257794 (PMC8550362; doi:10.1371/journal.pone.0257794)
Supplement: S1 Table — (DOCX) [file pone.0257794.s001.docx]

**Supporting Information**

**S1 Table. Difference in Prescribing Between Black and White Standardized Patients at Different Levels of IAT.**

|  | | | | |
| --- | --- | --- | --- | --- |
|  | OR | 95% CI | | p-value |
| -1 sd IAT (0.34) | 0.98 | 0.28 | 3.37 | 0.970 |
| mean IAT (0.89) | 0.27 | 0.09 | 0.86 | 0.027 |
| +1 sd IAT (1.44) | 0.08 | 0.01 | 0.48 | 0.006 |

*IAT=Implicit Association Test, sd=standard deviation, OR=odds ratio,*

*CI=confidence interval*
