# Supplementary material for: Is physician implicit bias associated with differences in care by patient race for metastatic cancer-related pain?
Source: PLoS One. 2021 Oct 27;16(10):e0257794. doi: 10.1371/journal.pone.0257794 (PMC8550362; doi:10.1371/journal.pone.0257794)
Supplement: S2 Table — (DOCX) [file pone.0257794.s002.docx]

**Supporting Information**

| **S2 Table. Results Based on Full IAT, Non-Pain IAT and Pain IAT.** | | | |
| --- | --- | --- | --- |
| **Variables** | **Full IAT** | **Non-Pain IAT** | **Pain IAT** |
| **Descriptive Statistics** |  |  |  |
| Alpha | 0.74 | 0.72 | 0.68 |
| Mean (SD) for Score | 0.89 (.06) | 0.86 (0.08) | 0.96 (0.07) |
| **Race x IAT  Parameter Estimate (95% CI***)* |  |  | |
| Opioid prescribing (OR) | **0.113 (*.064 - .783)**** | 0.355 (0.083-1.530) | 0.435 (0.91 - 2.11) |
| Patient centered pain talk (β) | **-4.163 (-8.113 - -.211)*** | -2.640 (-4.615 - 0.486) | -3.132 (-6.345 - 0.082) |
| Patient centered prognosis (β) | -.727 (-1.681 - .228) | -0.429 (-1.111 - 0.249) | -0.565 (-1.338 - 0.208) |
| Routine pain assessment (β) | -1.450 (-4.638 - 1.720) | 0.389 (-1.447 - 2.214) | -0.388 (-1.447 - 2.215) |
| Use of I-Statements (β) | -0.156 (-.757 - .445) | -0.357 (-0.741 - 0.027) | -0.147 (-0.571 - 0.277) |
| Physician Cut offs (OR) | 2.131 (.435 - 10.446) | 3.469 (0.843 - 14.276) | 1.139 (0.246 - 5.272) |
| Physician % Words (β) | -0.073 *(*-.150 - .004) | -0.0153 (-0.067 - 5.228) | -0.029 (-0.081 - 0.022) |

*IAT=Implicit Association Test, SD=standard deviation, OR=odds ratio, CI=confidence interval, β= regression coefficient*
